# Environmentally Friendly Polymer Compositions with Natural Amber Acid

**DOI:** 10.3390/ijms22041556

**Published:** 2021-02-04

**Authors:** Malgorzata Latos-Brozio, Anna Masek

**Affiliations:** Faculty of Chemistry, Institute of Polymer and Dye Technology, Lodz University of Technology, Stefanowskiego 12/16, 90-924 Lodz, Poland; malgorzata.latos@p.lodz.pl

**Keywords:** biodegradable polymers, aliphatic polyesters, amber acid

## Abstract

Few scientific reports have suggested the possibility of using natural phenolic acids as functional substances, such as stabilizers for polymeric materials. The replacement of commercial stabilizers in the polymer industry can be beneficial to human health and the environment. The aim of this study was to obtain biodegradable composition of polylactide (PLA) and polyhydroxyalkanoate (PHA) with natural amber (succinic) acid. The materials were subjected to controlled thermooxidation and solar aging. The research methodology included thermal analysis, examination of surface energy, mechanical properties and spectrophotometric analysis of the color change after aging. The samples of aliphatic polyesters containing from 1 to 2 parts by weight of succinic acid were characterized by increased resistance to oxidation (DSC analysis). Natural acid, preferably at a concentration of 1–1.5 parts by weight, acted as a stabilizer in the polymer compositions. On the other hand, materials that had amber acid above 2 parts by weight added were more susceptible to oxidation (DSC). They also showed the lowest aging coefficients (K). The addition of acid at 2.5–4 parts by weight caused a pro-oxidative effect and accelerated aging. By adding amber acid to PLA and PHA, it is possible to design their time in service and their overall lifetime.

## 1. Introduction

Nowadays, environmentally friendly and biodegradable polymer compositions are an important aspect of scientists’ research. Particularly noteworthy are polymers of natural origin, such as materials made of plant starch (e.g., polylactide), bacterial polymers (polyhydroxyalkanoates), cellulose, and compositions based on shellac, zein and other pro-ecological ingredients [1,2,3,4,5].

Polymer materials used in industrial practice practically always contain processing additives that give the finished products the expected properties. Stabilizers are usually one of the most important functional additives used in plastics processing. Stabilizers and their mixtures should protect plastics against the effects of various factors that lead to degradation of polymeric materials. Increased temperature, shear forces, UV radiation and the amount of available oxygen are the main factors determining degradation during processing and use of polymers. Typical stabilizers for polymers are hindered phenolic antioxidants, as well as compounds responsible for the decomposition of hydroperoxides, such as secondary stabilizers of the phosphorus or sulfur type [6,7].

Several years ago, the scientific literature raised questions about the effects of traditional phenolic stabilizers on human health and the environment, however, no alternative solution has yet been proposed in the industry. As a result, the possibility of using natural antioxidants as potential stabilizers for polymers is becoming more and more popular [8,9,10]. In the plastics industry, only natural α-tocopherol (vitamin E) is used on a large scale. The effectiveness of this compound as a processing stabilizer has been thoroughly investigated and described by Al-Malaika [11,12,13]. Vitamin E showed a better stabilizing effect on polyolefins than the synthetic antioxidants Irganox 1010 and Irganox 1076. Despite the high price, α-tocopherol is used to stabilize polyethylene medical devices [11,12,13]. Moreover, compounds of natural origin that have been proposed in the scientific literature as stabilizers for polymer materials include carotenoids, polyphenolic compounds (flavonoids, curcuminoids, phenolic acids) and phenolic polymers (lignin, tannin) [14,15,16,17,18,19,20,21,22,23].

One of the groups of natural antioxidants described for the stabilization of polymeric materials is acids of biological origin. Only a few attempts to use phenolic acids as stabilizers of polymeric materials have been described in the literature. The free radical scavenging mechanism of these polyphenols is based, as in the case of flavonoids, on the donation of a hydrogen atom from a phenolic hydroxyl group, but the efficiency of the reaction is strictly dependent on the chemical structure of the compound. For example, gallic acid acted as a pro-oxidant in the presence of transition metals, while chlorogenic acid was one of the most effective free radical scavengers [24,25,26,27]. Most phenolic acids have limited thermo-oxidative stability and poor solubility in nonpolar polymers, which may result in poor polymer stabilization efficiency [28]. Despite the above-mentioned disadvantages, the greatest advantage of phenolic acids is the presence of carboxyl groups in their structure, which are characterized by high reactivity. Carboxyl groups can take part in chemical reactions, allowing derivatives of these acids to be obtained with an increased number of phenolic hydroxyl groups and stronger antioxidant activity, as well as better solubility and higher thermal stability. An example of a phenolic acid modification reaction is esterification [29].

The aim of this study was to obtain pro-ecological, biodegradable polyester materials containing biological antioxidant–succinic acid. Succinic acid (known also as butanedioic acid, 1,2-ethanedicarboxylic acid and amber acid) occurs in nature as such or in various forms of its esters. In the environment, succinic acid is present in amber [30]. For example, Baltic amber contains about 3–8% of succinic acid, mainly in the external weathered layer or “amber cortex”. Baltic amber (succinite) is a fossil resin that was formed in natural conditions 45 million years ago. Despite the many processes that the resin underwent when it changed into amber, it still remains in the fossilization stage, i.e., oxidation and polymerization processes [31]. Succinic acid is utilized as a precursor to polymers, resins and solvents. In addition, this compound is used in the food industry as a food additive (acidity regulator) and dietary supplement [32]. Succinic acid has an antioxidant effect [33,34], and therefore, in this manuscript, it is proposed as an environmentally and consumer friendly stabilizer to control the lifetime of biodegradable aliphatic polyesters. Such application of this natural acid in polymeric materials has not been described in the literature. The combination of biodegradable polymers and succinic acid allows degradable polymer compositions to be easily obtained, for dedicated use as packaging materials. Thanks to the addition of natural acid, it is possible to control the overall life time and service life of the polymeric materials obtained.

## 2. Results and Discussion

**Figure 1:**
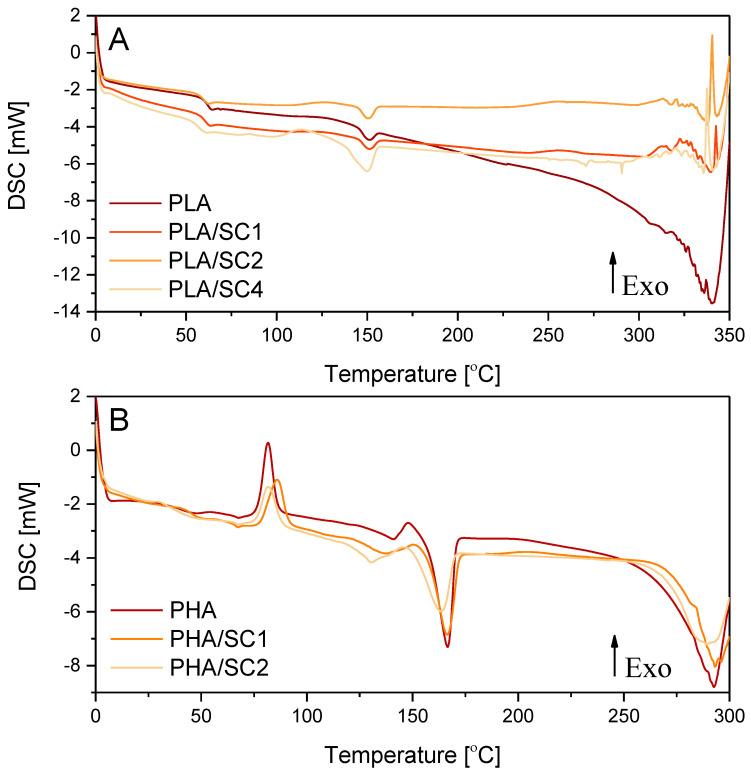
DSC thermograms of PLA (**A**) and PHA (**B**) samples with succinic acid (SC).

The study was initiated by analyzing the temperatures of the polymeric sample phase changes before aging. Table 1 and Figure 1 shows the results of differential scanning calorimetry (DSC) of compositions PLA and PHA with succinic acid (SC) added at different concentrations (1.0, 2.0 and 4.0 parts by weight).

The addition of succinic acid (SC) to PLA and PHA did not significantly change the temperature ranges of the sample phase changes: glass transition temperature (T_g_), crystallization temperature (T_cc_) and melting temperature (T_m_). The DSC curves of the PHA samples had two peaks corresponding to the melting of the crystalline phase, which is typical for this polymer. Therefore, there are two values, T_m_ and ΔH_m_, in Table 1 for a single sample.

The oxidation temperature is the temperature at which an exothermic oxidation peak appears on the DSC curve. Table 1 gives the initial (onset) temperatures of the oxidation peaks. Compositions of PLA and PHA with succinic acid were characterized by a clearly elevated oxidation temperature T_o_. The samples of the PLA/succinic acid composition containing 1–2 parts by weight of the natural acid had a T_o_ higher than the reference sample PLA by about 14 °C. The PHA/succinic acid samples containing 1–2 parts by weight of acid and were characterized by an oxidation temperature 41.7 °C and 43.6 °C higher, respectively. The clearly higher oxidation temperatures (T_o_) of the materials containing PLA, PHA and succinic acid (1–2 parts by weight) indicated increased resistance of the samples to oxidation, and thus increased resistance to the factors causing oxidation.

In the case of PLA and PHA samples containing more than 2.0 parts by weight of succinic acid, no oxidation peaks were recorded on the DSC thermograms, because the samples degraded intensively and only signals indicating the decomposition of the materials were obtained. The decomposition of PLA, PLA/SC1 and PLA/SC2 was observed at temperatures of 300–340 °C, while, at a higher acid concentration, decomposition started at a lower temperature of approximately 250 °C. The onset of decomposition of the extrudates made of PHA and PHA with 1–2 parts by weight of succinic acid was recorded at temperatures of 270–280 °C. Due to the lower oxidation temperature of PHA (199.2 °C) compared to PLA (226.5 °C) and very intensive material decomposition, DSC analyses of PHA samples containing more than 2.0 parts wt. succinic acid was not performed. Analysis could have damaged the DSC analyzer. Too high a concentration of succinic acid (above 2.0 parts by weight) may cause a pro-oxidative effect and accelerate the decomposition of polymeric materials. In samples containing more than 2 parts by weight of amber acid, there may be a high concentration of active groups that generate free radicals that can accelerate the aging process. Unfavourable phenomena (such as irritating, unpleasant odours), which may indicate the pro-oxidative effect of a higher concentration of succinic acid and decomposition of the samples, were already observed during the extrusion of samples containing over 2.0 parts by weight of a natural additive.

The addition of succinic acid at a concentration of up to 2.0 parts by weight to the PLA and PHA polymers had a stabilizing effect on the polymer. However, after introducing more than 2 parts by weight of the acid, the pro-oxidative effect of the natural compound resulted in accelerated and very intensive decomposition of polymeric materials.

A similar antioxidant and pro-oxidative effect of natural compounds was found for carotenoids added to polymers [35,36]. Carotenoids effectively inhibit the oxidation of polymers and effectively scavenge alkyl and alkoxy radicals at low oxygen concentrations. However, studies have shown that the addition of β-carotene can change its antioxidant effect so it becomes a pro-oxidant. This natural compound acts as a pro-oxidant at elevated temperatures and high oxygen concentrations, because its auto-oxidation becomes a thermodynamically favoured reaction [35]. Carotenes completely lose their stabilizing properties at increased oxygen concentrations and become pro-oxidants [36].

The addition of 1.0–2.0 parts by weight of succinic acid to polyesters seems to be the most advantageous in terms of antioxidant and stabilizing effect. Therefore, in subsequent tests, the flammability and changes in the elemental composition (oxygen content after ageing) of the selected PLA samples (PLA/SC1, PLA/SC1.5), containing 1.0 or 1.5 parts by weight of natural acid, were analyzed.

The PLA and PLA/SC1 samples were subjected to microscale combustion calorimetry. Heat release rate is a material flammability parameter. The heat release rate (HRR) was determined in the tests. The rate of heat release of polymeric materials is directly related to the rate of their weight loss during heating and is a function of the amount of heat delivered from the flame to the non-burning surface of the material. The heat release rate HRR for reference PLA was 1331 W/g and for the sample with succinic acid PLA/SC1 1172 W/g. The polyester containing 1.0 part by weight of amber acid was therefore characterized by a lower HRR. The rate of heat release depends on the degree of polymer carbonization after the combustion process. The addition of succinic acid to PLA may cause the formation of a char layer on the polymer surface during combustion. For polymeric materials forming a char layer as a result of combustion, the HRR value is lower. The reason for this is the protective role of the foamed coke layer, which under normal weather conditions is non-flammable and prevents free heat exchange and oxygen transport to the deeper layers of the burning material. According to the literature, natural compounds from the polyphenols group have good charring properties. In particular, tannins are characterized by an excellent charring capacity and can be used in flame retardant applications [37,38,39]. On the basis of the results obtained, it can be concluded that natural succinic acid, like polyphenols and tannins, has charring properties and reduces the flammability of polyesters.

**Figure 2:**
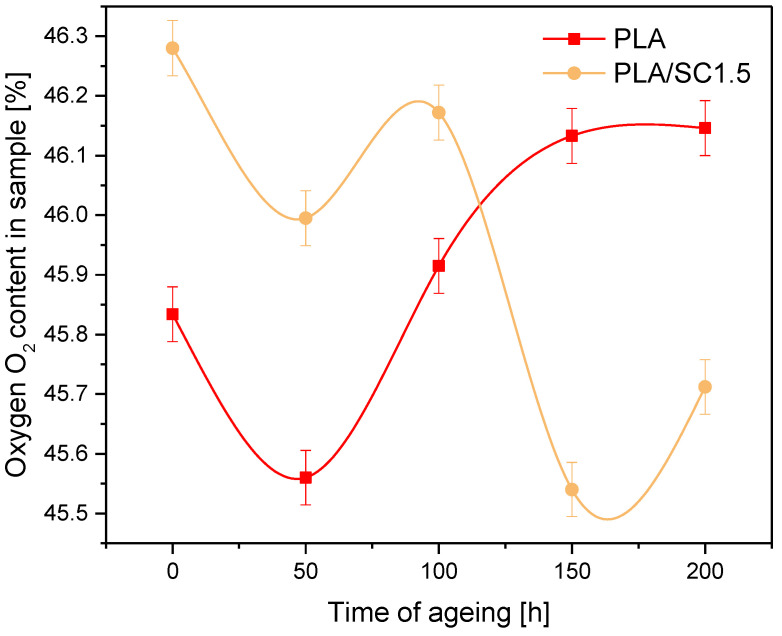
Oxygen content in PLA and PLA/Succinic acid (SC) 1.5 samples during solar aging.

The PLA/SC1.5 samples containing the amber acid concentration inducing antioxidant protection of the materials were subjected to elemental analysis. The changes of oxygen content in the composition during solar aging lasting 50, 100, 150 and 200 h were examined. The test results were compared with the results for the reference PLA without the natural additive (Figure 2).

The unaged PLA/succinic acid 1.5 sample had a higher oxygen content than the PLA reference sample. This seems obvious, due to the introduction to the polyester of an additional source of oxygen in the form of natural acid. From 100 h to 200 h of solar aging, an increase in the oxygen content in the PLA sample was found, as well as a decrease in the oxygen content in the PLA/succinic acid 1.5 material. In the PLA sample with acid, there was lower solubility and oxygen diffusion. The degradation processes during solar aging may cause detachment of substituents or breakage of C–C and C–H bonds in the main chain, with simultaneous formation of carbonyl, peroxide and hydroxyl groups. The appearance of carbonyl, peroxide and hydroxyl groups, accompanying the degradation of the polymer, may cause an increase in oxygen content in PLA samples that were not protected by natural acid. For a polyester sample containing succinic acid, a natural substance, it can act as an antioxidant. Succinic acid can stabilize the polymer as a free radical scavenger and also as an antioxidant preventing or delaying polymer oxidation in reactions with RO^●^ radicals, peroxide radicals (ROO^●^), and also hydroperoxide groups (R-OOH). As a result of the stabilizing effect of amber acid, the oxygen content in the samples after solar aging was lower than in the reference sample. The research confirmed the stabilizing and antioxidant effect of the addition of succinic acid up to 2.0 parts by weight to aliphatic polyesters.

**Figure 3:**
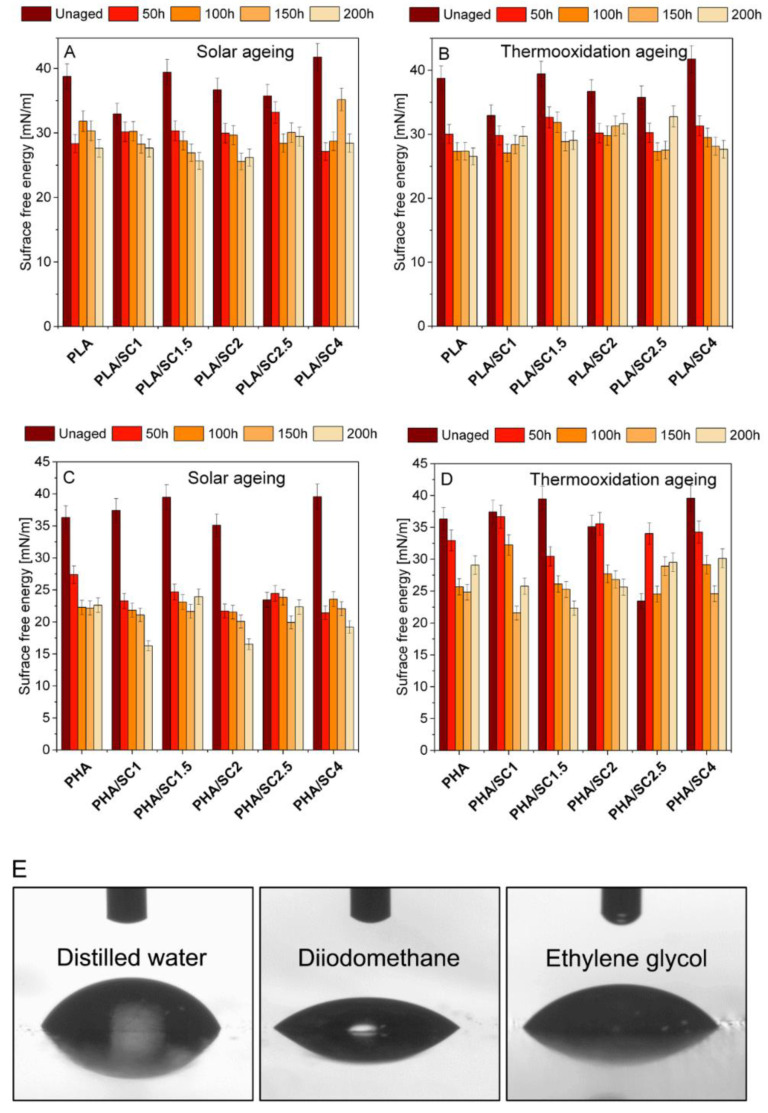
The changes in PLA (**A**,**B**) and PHA (**C**,**D**) with succinic acid (SC) surface free energy after solar and thermooxidation aging; drop of distilled water, diiodomethane and ethylene glycol on PLA before aging (**E**).

In the next step of the research, changes in the surface energy of PLA and PHA materials after solar and thermooxidation aging were analyzed (Figure 3). Surface free energy is one of the thermodynamic functions describing the state of equilibrium between atoms in the surface layer of materials. PLA and PHA are hydrophilic materials susceptible to aging. As a result of degradation of polymers, the free energy of the surface should increase. Due to the aging processes taking place in the structure of the materials, it is more difficult to create a unit of surface, as is apparent from the definition of the surface energy. Migration of antioxidants to the surface of polymeric materials can also result in increased SEP.

Figure 3 summarizes values of surface free energy after solar and thermooxidation aging. For the unaged PLA samples, the surface free energy was 32.95–41.75 mN/m—the lowest value for the PLA/SC1 sample and the highest for PLA/SC4. The surface free energy decreased after thermooxidation and solar aging compared to unaged polyester, regardless of the amber acid concentration. The decrease in surface energy after aging may be due to poor dissolution of the acid in the polymer, low miscibility of the natural additive, or migration of the acid to the surface, resulting in reactions of succinic acid functional groups with the measuring liquids. Analogous results were obtained for the PHA polymer. The aged samples were characterized by lower surface energy values than the reference samples before aging.

**Figure 4:**
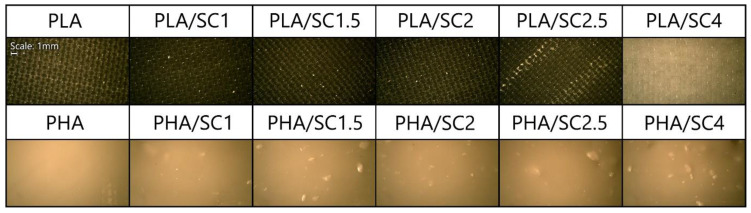
Visualization of PLA and PHA samples with succinic acid. Photographs taken with an optical microscope at 50× magnification.

The limited solubility and miscibility of succinic acid with selected polymeric materials resulted in obtaining heterogeneous samples, especially with the addition of succinic acid over 2.0 parts by weight. Figure 4 shows microscopic photos of the samples indicating their non-homogeneity. The photos of the samples were taken on a black background. Streaks of undissolved additive appeared in the transparent PLA samples after adding 2.5 parts by weight of succinic acid. The PLA/SC4 material, unlike the other PLA-based samples, was characterized by a milky-white color, which was the result of the highest content of succinic acid. In all samples of PHA with amber acid (especially in PHA/SC2, PHA/SC2.5 and PHA/SC4), small pieces of acid were visible. The morphology of the samples undoubtedly influenced the determination of the surface energy of the samples and the lack of typical trends in its changes due to aging. The functional groups of succinic acid that were located on the surface of the polymeric materials may react with the liquids used in measuring the contact angles. As a result of these chemical reactions, the surface energy of the samples after aging was lower than before the controlled degradation.

For the aged polymer materials, mechanical property tests are one of the basic determinations carried out in order to determine the influence of degradation factors on the mechanical strength of materials. In addition, mechanical tests are used to indirectly examine the effects of aging under given conditions and may be one of the sources of information about the decomposition processes taking place in the materials.

**Figure 5:**
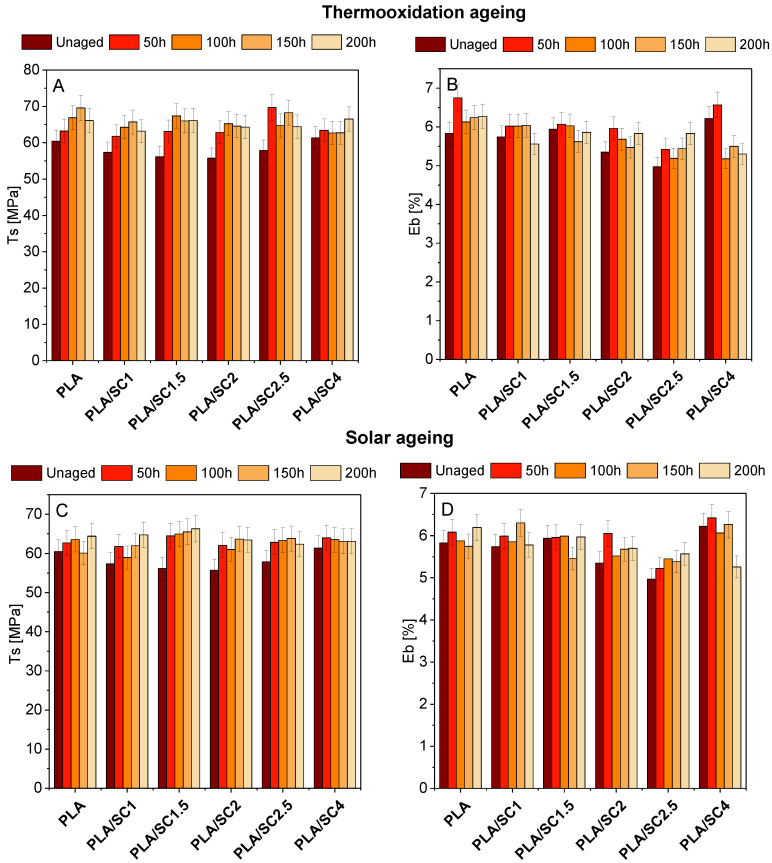
The changes in PLA mechanical properties (T_s_, the tensile strength, and E_b_, the elongation at break) before and after thermooxidation (**A**,**B**) and solar aging (**C**,**D**).

**Figure 6:**
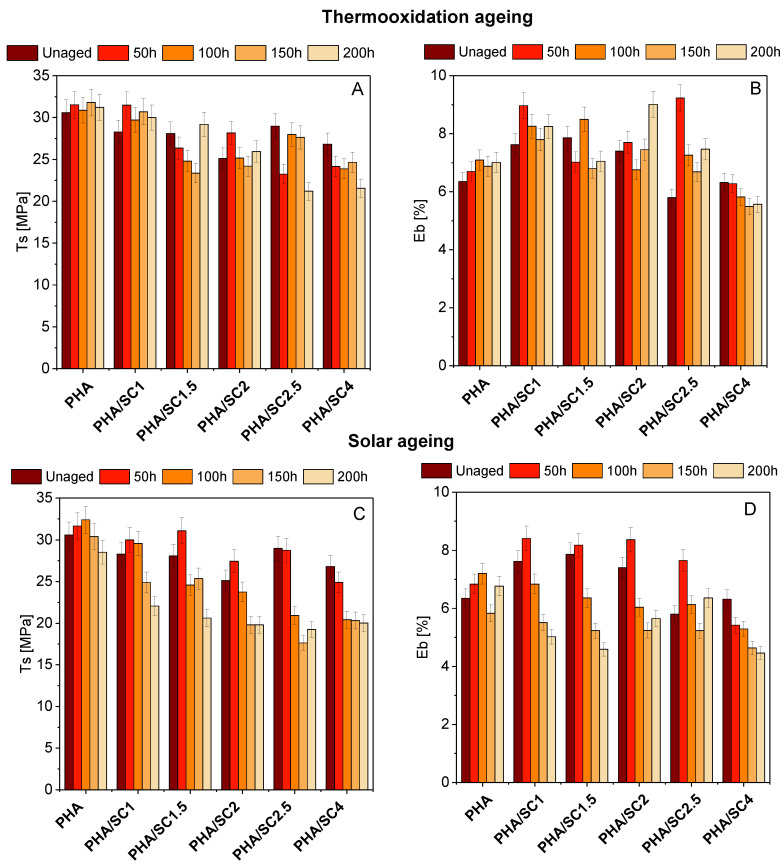
The changes in PHA mechanical properties (Ts, the tensile strength, and E_b_, the elongation at break) before and after thermooxidation (**A**,**B**) and solar aging (**C**,**D**).

Figure 5 and Figure 6 summarize the changes in PLA and PHA mechanical properties (T_s_, the tensile strength, and E_b_, elongation at break) before and after solar and thermooxidation aging. The addition of succinic acid to PLA slightly affected the mechanical properties of the materials. The tensile strength value of the reference PLA was 60.45 MPa, and for the samples with the natural addition it was 55.75–61.38 MPa. The elongation at break of the samples with acid was also close to the value of the reference sample (E_b_ PLA = 5.83%, E_b_ PLA/SC = 4.97–6.22%). After aging, especially after solar aging, an increase in the tensile strength was seen for the PLA sample and for materials containing amber acid. The elongation at break changed most clearly after thermooxidation aging for the PLA/SC4 sample. The tendency for the T_s_ value to increase may be due to the increase in crystallinity of PLA samples during aging. The aging/degradation of PLA is specific and starts with an increase in the crystallinity of the samples, resulting in higher values of the strength properties (T_S_). In the next stage of degradation, the crystalline phase content in the samples decreases and the mechanical properties (parameter T_s_) decrease [40,41]. The 200 h aging time may be too short for the degradation of the PLA material in which a decrease in the strength is observed.

As in the case of PLA polyester, the addition of succinic acid to PHA slightly changed the mechanical properties (Ts, E_b_) (Figure 6). More pronounced changes in strength were visible after aging. For PHA samples, for most materials, there is a slight increase in the Ts values after 50 h of thermooxidation and solar aging, and a decrease in strength properties was observed in the further stage of both aging procedures. The lowest Ts values after solar aging were obtained for samples PHA/SC2 (Ts = 19.81 MPa) and PHA/SC2.5 (Ts = 17.63 MPa), while for materials after thermooxidation aging, the lowest values of the Ts parameter were PHA/SC2.5 (Ts = 21.20 MPa) and PHA/SC4 (Ts = 21.56 MPa). Moreover, the Eb for PHA-based materials was also characterized by greater change compared to PLA samples. A clear decrease in elongation at break was seen for samples after solar aging. The decrease in the mechanical properties of PHA materials suggests a greater susceptibility of this polyester to aging compared to PLA.

**Figure 7:**
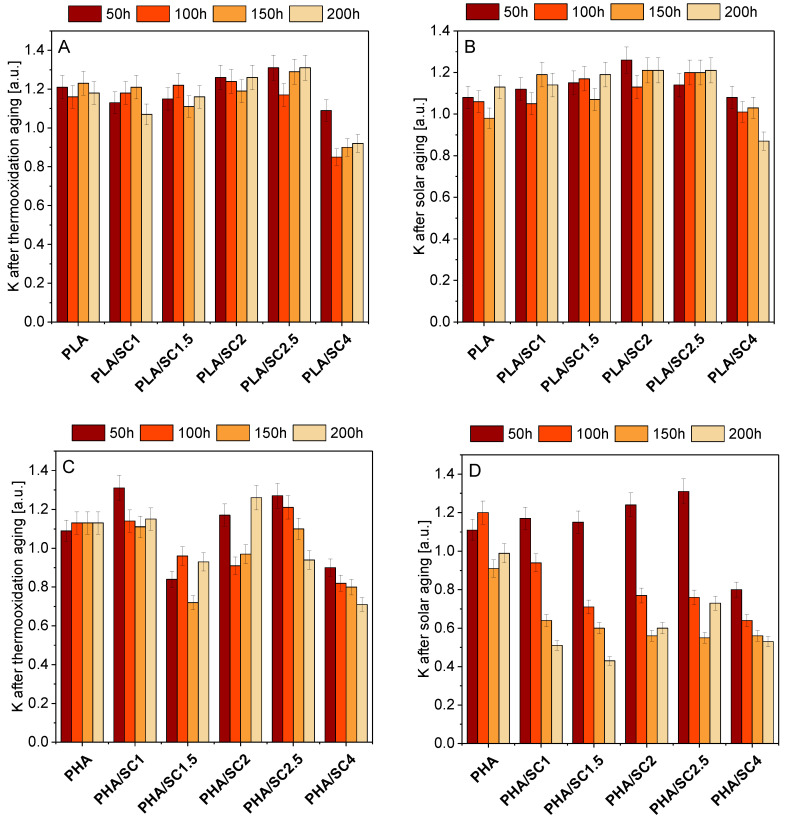
The aging coefficient (K) of PLA (**A**,**B**) and PHA (**C**,**D**) with succinic acid (SC) after thermooxidation and solar ageing. Resistance to degradation of samples: K = 1 resistant, K = 0 susceptible.

On the basis of static mechanical properties, the aging coefficients (K) of polymeric composition after thermooxidation and solar aging were determined (Figure 7). The aging coefficient characterizes the degree of material degradation. A value of K close to 0 meant that the sample was more susceptible to aging. A value of K close to 1 meant that the sample was resistant to degradation.

For PLA-based materials, significantly lower aging coefficients were found for samples containing the highest concentration of succinic acid (4.0 parts by weight of succinic acid). The PLA/SC4 sample was most susceptible to thermooxidation and solar aging. The addition of 4.0 parts by weight of amber acid to polylactide made the polyester more sensitive to degradation and, therefore, it could have a pro-oxidative effect. Similarly, the pro-oxidative effect of the addition of 4 parts by weight of succinic acid was also found on the basis of DSC analysis. PLA samples containing from 1.0 to 2.5 parts by weight of the natural additive showed better or comparable resistance to both types of aging as the reference material (greater or comparable aging coefficients). The antioxidant effect of succinic acid at 1.5 parts by weight in PLA was also confirmed on the basis of elemental analysis in the previously described studies.

Compared to PLA samples, materials made of PHA were more susceptible to degradation caused by thermooxidation and solar aging, as evidenced by lower K aging parameters. Solar aging caused degradation to a greater extent, which was confirmed by the lowest K factors. Samples were affected by more degrading factors during solar aging than during thermal aging, that is UV radiation in addition to elevated temperature.

The lowest values of the K parameter, considering the samples aged by both thermooxidation and solar, were obtained for material containing 4.0 parts by weight of succinic acid, therefore it can be concluded, similarly to PLA, that the addition of such an amount of amber acid to the polymer PHA may be a factor accelerating the aging.

The PHA samples being more prone to aging than PLA may be due to the morphology of the materials. Other studies by the authors (SEM analysis) [40,41] showed that PLA had a compact, smooth structure, while the PHA samples were porous. The higher susceptibility of PHA to degradation may be related to its high porosity, i.e., a larger contact surface of the sample with degrading factors and easier oxidation of the material.

**Figure 8:**
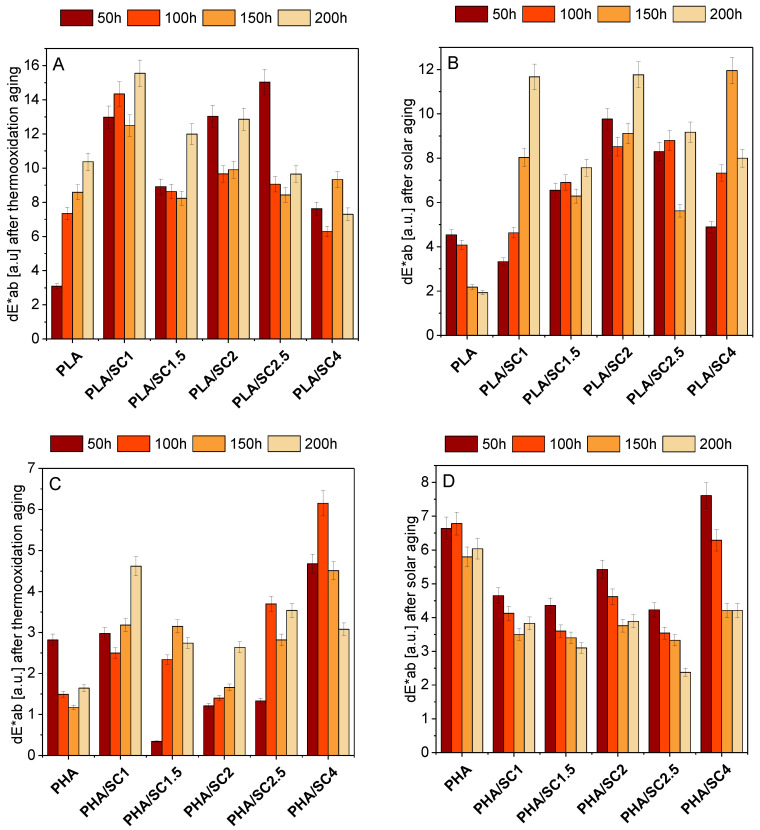
The changes in color of PLA (**A**,**B**) and PHA (**C**,**D**) with succinic acid (SC) after thermooxidation and solar aging.

The change of color of polymeric materials is the first sign of their degradation. Figure 8 shows the color changes of PLA and PHA samples after thermoxidation and solar aging. Statistically, the colors do not differ when dE*ab <1. When 1< dE*ab < 2, only an experienced observer can see the difference between the color of the samples. When the change in the color coefficient dE*ab is 2 < dE*ab < 3.5, the difference in color can be seen by the average observer. The range of 3.5 < dE*ab < 5.0 means that there is a distinct color difference, while in the dE*ab > 5 range, colors are perceived as completely different. The color change factor of PLA samples after thermo-oxidative and solar aging was from about 2 to about 16 [a.u.]. This means that the color change of the samples with the lowest dE*ab factor was visible to the average observer, while the color of the samples with the highest index was perceived as completely different in relation to the unaged sample. Among all materials based on PLA, the reference sample after solar aging showed the least color change. The samples containing succinic acid presented a distinct color change (dE*ab 3.5–12 [a.u.]) after ageing.

In contrast to PLA-based compositions, the PHA reference sample after solar aging showed the greatest color change (excluding PHA/SC4 after 50 and 100 h). The materials with natural acid had lower dE*ab coefficients than the sample standard. PHA samples after thermooxidation aging were characterized by the lowest color change of the two polyesters.

Generally, PLA polyester samples showed a greater color change compared to PHA samples. A stronger effect of the addition of succinic acid on the color change was found for the PLA compositions. This may be due to the transparency of the polymer and the incomplete solubility of the acid. Due to the clear color change of PLA samples (dE*ab > 5), amber acid can be used as an indicator of the aging time of this polyester. After aging, especially after solar aging, the PLA samples changed their color from transparent to milky white.

The service life and overall lifetime of polymeric materials depend on many factors, including functional additives such as stabilizers. The polymer matrix also plays an important role as it has intrinsic resistance to degradation under the influence of external factors. This manuscript focuses on determining the mechanisms of antioxidant and pro-oxidative action of succinic acid added to aliphatic biodegradable polyesters.

The reactivity of molecular oxygen, the basic element of the atmosphere, is very low and it does not attack polymers. Polymers are resistant to molecular oxygen attack because they have paired electrons with a net spin equal to zero. The reactions of free radicals or macro radicals accompany every oxidative degradation reaction with oxygen in the air.

If a free radical R^●^ or a macro radical meets an oxygen molecule, it immediately reacts with it, resulting in the formation of ROO^●^ peroxide radicals. Peroxide radicals easily detach the hydrogen atom from the same or another macromolecule, creating a hydroperoxide group (OOH). The hydroperoxide groups generated easily undergo thermal or photochemical dissociation, forming an oxide radical (RO^●^) and a hydroxyl radical (HO^●^). Free oxygen radicals (ROO^●^ and RO^●^) readily undergo recombination reactions, forming ROOR peroxy groups and ROR oxide groups in the polymer backbone. The peroxide groups in the polymer backbone easily undergo thermal or photochemical dissociation to form RO^●^ oxide radicals.

The oxidation of polymeric materials depends on the chemical and molecular structure of the polymer. Semicrystalline polymers are more difficult to oxidize than amorphous ones. Branched polymers oxidize faster than unbranched polymers. Saturated polymers oxidize more slowly than polymers containing double bonds. In addition, temperature and UV radiation accelerate the oxidation mechanisms of polymers [42,43], such as in the case of solar and thermo-oxidative aging.

In order to reduce the harmful effects of oxygen on polymeric materials, antioxidants are added to them. The mechanism of action of natural polyphenolic compounds, which determines the stabilization of polymers, is based on the transfer of hydrogen atoms and chelation of transition metal ions. Transition metal ions can catalyse polymer aging reactions. Polyphenolic compounds have the ability to react with alkoxy and peroxy radicals, as well as with transition metals. The stabilizing effect of polyphenolic acids is based on the mechanism of scavenging free radicals by donating a hydrogen atom from a phenolic hydroxyl group, as in the case of flavonoids [14].

Like the phenolic acids, the mechanism of free radical scavenging by succinic acid may be based on the donation of a hydrogen atom from OH groups. Moreover, carboxyl groups, characterized by high reactivity, can take part in chemical reactions allowing acid derivatives with an increased number of phenolic hydroxyl groups to be obtained, which can produce a stronger antioxidant activity.

As with other acids, such as gallic and ascorbic acid, succinic acid also showed both antioxidant and pro-oxidative effects. The pro-oxidative mechanism of the action of natural acids was probably due to the strong reduction force and poor ability to chelate metal ions [44].

Succinic acid added to polymers in a concentration above 2 parts by weight caused a pro-oxidative effect, accelerating aging. This may be due to the strong reducing force of the acid, or the auto-oxidation may, under certain conditions, be a thermodynamically favoured reaction. In order to fully understand the pro-oxidative and antioxidant effects of succinic acid, it may be helpful to perform electrochemical tests in an environment corresponding to that of polymer matrices.

## 3. Materials and Methods

### 3.1. Reagents

The pro-ecological polymeric materials utilized in this study were PLA—polylactide and P(3,4HB) polymer belonging to the PHA (polyhydroxyalkanoate) group. Polylactide (PLA), IngeoTM Biopolymer 4043D PLA, was produced by Nature WorksTM (Minnetonka, MN, USA). According to the manufacturer, the polymer had the following properties: T_g_ = 55–60 °C, Tm = 145–160 °C, and melt flow index MFI = 6 g/10 min. PLA is a biodegradable material produced from starch derived from various natural sources such as tapioca and corn. The second polymer used in the research, aliphatic polyester (PHA), was purchased from Simag Holdings LTD (Hong Kong, China). Polymeric materials PHA had the following properties: P(3,4HB) containing 12 mol% 4-hydroxybutyrate, the average fold was approximately 520 kDa, MVR = 15–20 g/10 min, (assay conditions: temperature 170 °C, nominal load 2.16 kg) and a density of 1.25 g/cm^3^. Polymers from the group of polyhydroxyalkanoates are synthesized by some bacteria as intracellular storage compounds.

**Figure 9:**
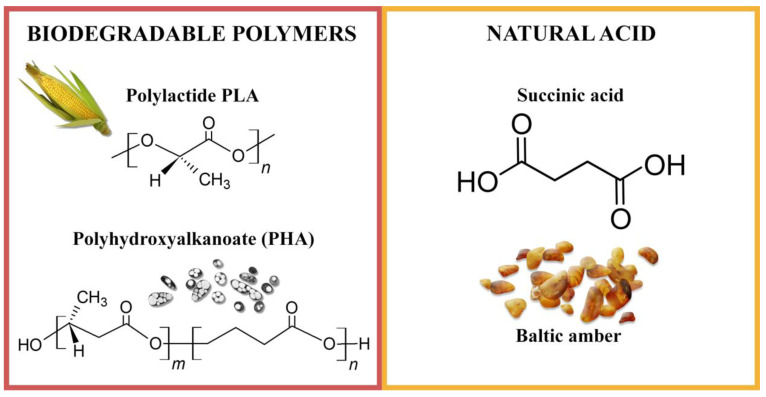
Structure and sources of biodegradable aliphatic polyesters and succinic acid.

Succinic acid (natural, ≥99%, *M*_W_ = 118.09 g/mol) was obtained from Sigma Aldrich (Steinheim, Germany). Figure 9 shows the structural formulas of the polymers and natural acid used.

### 3.2. Preparation of PLA and PHA Samples Containing Succinic Acid

PLA and PHA granulates were dried under the following conditions: time 12 h, temperature 50 °C. In next step, dried polyesters PLA and PHA were mixed with 1.0, 1.5, 2.0, 2.5 and 4.0 parts by weight of succinic acid (SC) and extruded by applying a laboratory extruder. For each 100 g of PLA and PHA polymer granules, 1, 1.5, 2, 2.5 and 4 g of succinic acid were added, respectively. Strip-shaped samples with a thickness of 1.6–1.8 mm were prepared. The following temperatures were used during the extrusion process for PLA: feed zone temperature 25 °C; cylinder zone temperature 190 °C; nozzle zone temperature 180 °C. For PHA, the temperatures were: feed zone temperature 25 °C; cylinder zone temperature 165 °C and nozzle zone temperature 160 °C. The screw rotation speed was 40 rpm and the extrusion pressure was 17 atm.

### 3.3. Controlled Aging of Polymeric Materials

Solar Aging: The polymeric compositions were aged in an Atlas SC340 MHG Solar Simulator (AMETEK, Inc., Berwyn, IL, USA) climatic chamber with an MHG 2500 W lamp. The exposure time consisted of 30 complete cycles of 24 h, each cycle divided into 20 h at an intensity of 1200 W/m2 and 4 h without exposure to solar radiation. Solar aging lasted 50, 100, 150 and 200 h at 60 °C and 70% humidity.

Thermooxidation Aging: The reference PLA and PHA samples and materials with succinic acid were exposed to air at an elevated temperature (70 °C) for 50, 100, 150 and 200 h in a heating chamber (Binder, Germany) with forced convection.

### 3.4. Measurement Methods

Differential scanning calorimetry (DSC): A Mettler Toledo DSC analyzer (Greifensee, Switzerland) was used for the measurements. The temperatures of the polymeric sample phase changes, i.e., the glass transition temperature (T_g_), cold crystallization temperature (T_cc_), melting temperature of the crystalline phase (T_m_) and oxidation temperature (T_o_) was determined. The heat (ΔH) accompanying the phase changes was also examined. Test samples (5 μg) were placed in 100-μL open, aluminium pans. The PLA and PHA materials were heated from 0 to 200 °C at a rate of 20 °C/min under an argon atmosphere. After 10 min at 200 °C, the samples were cooled to 0 °C. At that time, the gas was switched from argon to air (flow rate 50 mL/min), and in the next step the samples were heated to 350 °C.

Microscale combustion calorimetry: Microscale combustion calorimetry (MCC) tests were made on 2.5 mg samples using a microcalorimeter (Fire Testing Technology Limited, East Grinstead, UK). The temperature of the pyrolyser was 750 °C, and that of the combustor 900 °C. During the measurements, the following parameters were recorded: Maximum heat emission rate (HRRmax), total heat emitted (THR), and heat capacity. The samples were heated with a linear temperature program. The volatile thermal degradation products were swept from the pyrolysis chamber by an inert gas and combined with excess oxygen in a tubular furnace at a temperature of 900 °C. This ensured the complete combustion (oxidation) of the fuel. Products of combustion (CO_2_, H_2_O and acid gases) were scrubbed from the gas stream. The transient heat release rate was computed from the measured flow rate and oxygen concentration after correcting for flow dispersion. The maximum (peak) value of the MCC heat release rate (HRRMAX) was normalized to the initial sample mass. Heat release rate is a material flammability parameter, measured in units of heat release capacity (J/g K). This parameter depends only on the chemical composition of the material and is proportional to the burning rate of the sample in a fire.

Elemental analysis: Using a NICUBE analyzer (Elementar Analysensysteme GmbH; Langenselbold, Germany), the relative oxygen content of the samples of the polymeric materials was determined before aging and after solar aging for 50, 100, 150 and 200 h. The method of pyrolytic decomposition against carbon was used for the determinations.

Surface free energy (SEP): The test was made for the PLA and PHA samples before and after solar and thermooxidation aging. The OEC 15EC goniometer (DataPhysics Instruments GmbH, Filderstadt, Germany) was used to measure surface free energy. The determination of the surface energy was made on the basis of the measurement of the contact angle for liquids with different polarity: distilled water, diiodomethane and ethylene glycol. On each of the three tested samples of one material, 10 contact angles were made for three measuring liquids.

The free surface energy was calculated by the Owens, Wendt, Rabel and Kaelble (OWRK) method. The SCA 20 software was used for the calculations. Polar and disperse contributions to the surface energy and surface tension were combined by forming the sum of both parts, leading to Equations (1) and (2):

(1) $$\sigma_{l}=\sigma_{l}^{d}+\sigma_{l}^{p}$$

(2) $$\sigma_{S}=\sigma_{S}^{d}+\sigma_{S}^{p}$$

where $\sigma_{l}^{d}$ and $\sigma_{l}^{p}$ describe the disperse and polar parts of the liquid, while $\sigma_{s}^{d}$ and $\sigma_{s}^{p}$ stand for the respective contributions of the solid.

Aging coefficient (K): On the basis of static mechanical properties, the aging coefficient (K) of th epolymeric composition base on PLA and PHA, after solar and thermooxidation aging, was determined. The aging coefficient (K) was computed from Equation (3).

(3) $$K=\;\frac{\left(T_{s}\times E_{b}\right)\;after\;ageing}{\left(T_{s}\times E_{b}\right)\;before\;ageing}$$

where: *T_S_*—tensile strength [MPa], *E_b_*—elongation at break [%].

The values of the aging factor K are in the range of 0–1 [a.u.] The value above this range (above the value 1 [a.u.]) results from the measurement uncertainty of the device for determining mechanical properties.

Mechanical properties of polymeric compositions were done using a Zwick Roell Z005 test machine (Zwick Roell, Ulm, Germany). Six control samples were cut out from extruded strips with a thickness of 1.6–1.8 mm and length of 150 mm. These samples were used for mechanical tests. The measurement conditions were: a preload of 0.1 N and a test speed of 50 mm/min.

Change of color: A spectrophotometer CM-3600d (Konica Minolta Sensing, Osaka, Japan) was used for color testing. Color measurements were performed before and after solar and thermooxidation ageing to determine the color change of the samples. The result of the examination is the color as described in the CIE-Lab space and the color in a system of three coordinates: L, a and b, where L is the lightness parameter (maximum value of 100, representing a perfectly reflecting diffuser, minimum value of zero representing the color black), a is the axis of red-green and b is the axis of yellow-blue. The a and b axes have no specific numerical limits. The change of color, dE×ab, was computed according to Equation (4).

(4) $$dE\times ab=\sqrt{(\Delta a^{2})+\left(\Delta b^{2}\right)+\left(\Delta L^{2}\right)}$$

For the change of color examination, tree samples of each type of the test materials were prepared for each time interval of ageing. Spectrophotometric measurements were done at five measuring points on each of the tree control/reference samples.

Optical microscopy: The photos were taken with a Leica optical microscope using a ring light to illuminate the samples. The analysis and processing of the photos was performed using Optaview software.

## 4. Conclusions

The results presented suggest that succinic acid can be successfully used as an antioxidant and pro-oxidant substance for biodegradable polyesters. As a result of adding succinic acid in a concentration of 1–2 parts by weight to aliphatic polyesters, an increase in the oxidation temperature of the samples (at least by 14 °C for PLA and 41 °C for PHA) was observed, which allows materials to be obtained with higher resistance to oxidation and with extended lifetime. At a concentration of 1–2 parts by weight, amber acid can be used as a stabilizer for polymeric materials. On the other hand, the introduction of succinic acid to PLA and PHA in an amount exceeding 2.0 parts by weight resulted in obtaining materials characterized by accelerated oxidation, as evidenced by DSC analysis and aging factors. Such polymer compositions have potential use as disposable packaging materials with a very short lifetime. Moreover, the materials obtained, especially PLA, showed a clearly visible change of color after thermooxidation and solar aging. A change in the color of polymeric materials may indicate their lifetime. Due to the addition of an appropriate amount of succinic acid, the life time of polyester materials can be controlled, i.e., polymer compositions with increased resistance to aging or materials with accelerated aging can be obtained.

## Figures and Tables

**Table 1:** The DSC analysis of the PLA and PHA samples with succinic acid (SC).

SC Sample Composition in Parts by Weight	Tg [°C]	Tcc [°C]	ΔHcc [J/g]	Tm [°C]	ΔHm [J/g]	ΔHo [J/g]	To [°C]
PLA	58.5	108.8	4.7	145.4	3.1	22.5	226.5
PLA/SC1	57.3	106.8	3.4	145.1	3.2	2.5	240.9
PLA/SC2	56.5	109.5	5.6	144.9	4.4	10.4	239.8
PLA/SC4	52.6	100.5	10.7	138.1	13.0	-	-
PHA	36.7	76.7	15.3	(1) 127.8 (2) 156.6	(1) 6.5 (2) 33.9	9.6	199.2
PHA/SC1	41.4	77.7	14.4	(1) 124.5 (2) 157.1	(1) 6.5 (2) 27.6	1.6	240.9
PHA/SC2	37.5	75.7	11.5	(1) 121.7 (2) 150.9	(1) 8.9 (2) 23.9	0.8	242.8
PHA/SC4	*						

T_g_, glass transition temperature, T_cc_, cold crystallization temperature, ΔH_cc_, enthalpy of cold crystallization, T_m_, melting temperature, ΔH_m_, enthalpy of melting, ΔH_o_, enthalpy of oxidation, T_o_, oxidation temperature. * Due to the very intense material decomposition, DSC analysis was not performed for PHA samples containing more than 2 parts by weight of succinic acid.

## Data Availability

Not applicable.
